# Reduced expression of circRNA hsa_circ_001888 in gastric cancer and its clinical significance

**DOI:** 10.1002/jcla.23953

**Published:** 2021-08-16

**Authors:** Peina Shi, Haojun Song, Xiaoyun Ding

**Affiliations:** ^1^ The Gastroenterology Department of Ningbo First Hospital Ningbo China; ^2^ The Gastroenterology Department of Ningbo Yinzhou No. 2 Hospital Ningbo China

**Keywords:** biomarker, circular RNA, diagnosis, gastric cancer, hsa_circ_001888

## Abstract

**Background:**

Circular RNAs (circRNAs) are a novel family of endogenous RNAs. Recent studies have demonstrated that circRNAs are potential novel biomarkers for diagnosing cancers. However, little is known about the role of circRNAs in gastric cancer (GC). This study aimed to identify the relationship between GC and a new circRNA named hsa_circ_001888.

**Methods:**

Hsa_circ_001888 expression levels were measured by quantitative reverse transcription‐polymerase chain reaction (qRT‐PCR) in GC cell lines, tissues, and plasma samples. Then, the associations between the expression level of hsa_circ_001888 and the clinicopathological features of patients with GC were further investigated. A receiver operating characteristic (ROC) curve was generated to evaluate the diagnostic value of hsa_circ_001888.

**Results:**

In this study, hsa_circ_001888 was first found to be significantly downregulated in GC cell lines (AGS and MKN‐45), tissues, and plasma samples compared to control samples. Clinicopathological features showed that the expression of hsa_circ_001888 in GC tissues was associated with differentiation and in GC plasma linked with serum CEA and CA19‐9 levels. The areas under the ROC curves of hsa_circ_001888 in tissues and plasma were 0.654 and 0.66, respectively.

**Conclusions:**

Hsa_circ_001888 may serve as a potential biomarker in the diagnosis of GC and may be involved in GC development.

## Graphical abstract

This study aimed to identify the relationship between gastric cancer (GC) and a new circRNA named hsa_circ_001888. The results indicated that hsa_circ_001888 was significantly downregulated in GC cell lines, tissues and plasma samples compared to control samples. Hsa_circ_001888 may serve as a potential biomarker in the diagnosis of GC with high degrees of specificity, sensitivity and accuracy.

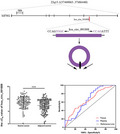

## 1 INTRODUCTION

Gastric cancer (GC) remains one of the most common gastrointestinal malignancies worldwide, with a high mortality rate and a poor survival rate.^1^, ^2^ It is the fourth most common cancer, the third leading cause of cancer‐related death worldwide, and the most frequently diagnosed gastrointestinal cancer among East Asian populations.^1^ Due to the lack of characteristic clinical symptoms and reliable biomarkers, most patients with GC are not diagnosed early in the course of the disease. Although the various diagnostics and treatments have been improved, the 5‐year survival rate of GC remains low since it is often diagnosed at a late stage.^3^ Therefore, exploring a novel type of ideal cancer biomarker may help to improve the early diagnosis of GC.

Circular RNAs (circRNAs) are a novel family of endogenous RNAs with characteristics such as diversity, abundance, conservation and stability.^4^, ^5^ Unlike linear RNAs, circRNAs form covalently closed‐loop structures without 5′ end caps or 3′ polyadenylated tails.^5^, ^6^ Recent studies demonstrated that circRNAs have several critical functions in the pathogenesis of human disease and can be potential novel biomarkers in diagnosing cancers. Increasing evidence has shown that circRNAs are associated with a wide variety of cancers, such as colorectal cancer, laryngeal cancer and breast cancer, among other cancers.^7^ However, the role and function of circRNAs in GC remains largely vague.^8^, ^9^

**FIGURE 1:**
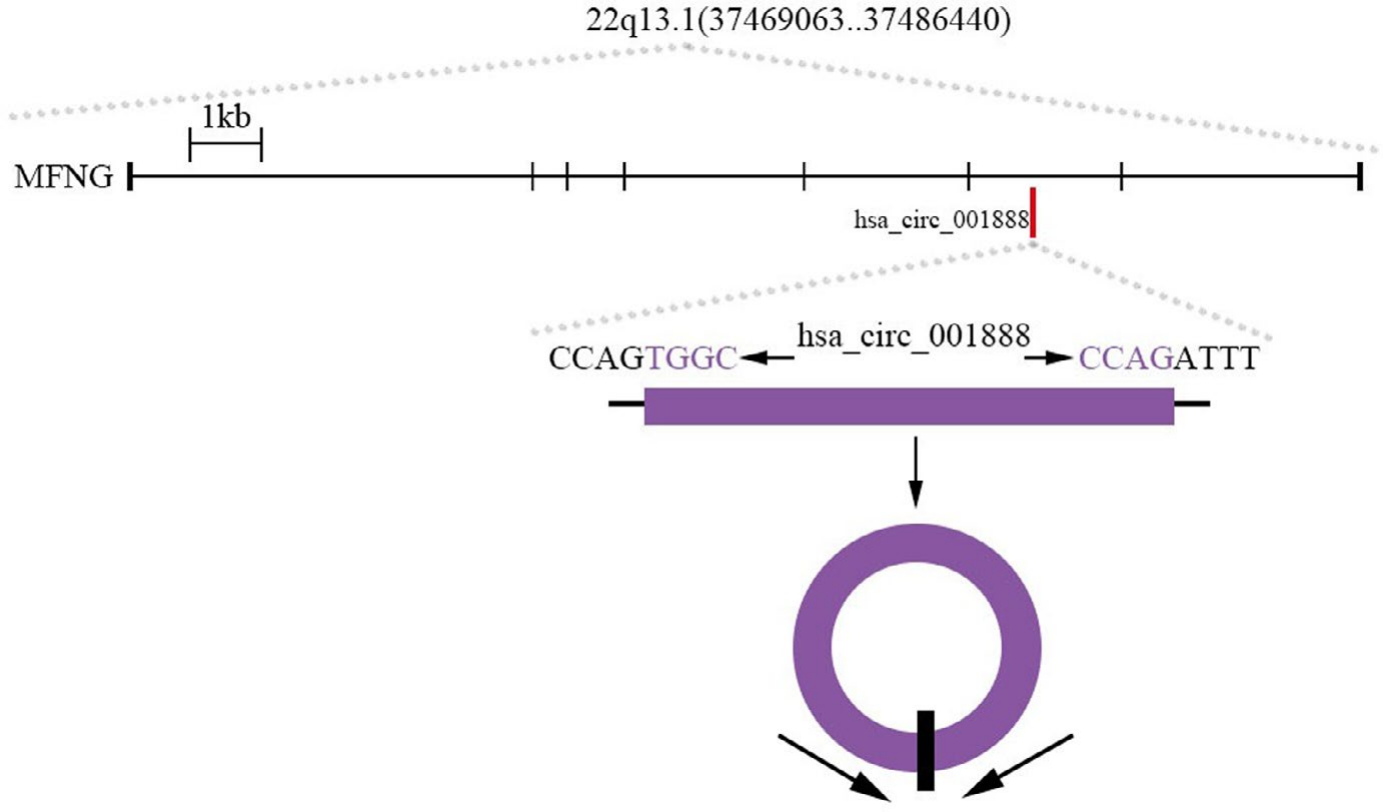
The genomic loci of the MFNG gene and hsa_circ_001888

This study aimed to identify the association between GC and a new circRNA named hsa_circ_001888 (Alians, hsa_circ_0001228; http://circbase.org/). Its gene is located at chr22: 37868480‐37870715, and its associated gene symbol is MFNG (O‐fucosylpeptide‐3‐beta‐N‐acetylglucosaminyltransferase). Its spliced sequence length is 2235nt (Figure 1). In this study, we first verified that hsa_circ_001888 expression levels were significantly different in GC cell lines, GC tissues, and GC plasma samples. Then, the relationship between hsa_circ_001888 expression levels and the patient clinicopathological factors was further investigated. Our data indicated that hsa_circ_001888 may serve as a novel potential biomarker in the diagnosis of GC.

## 2 MATERIALS AND METHODS

### 2.1 Patients and sample collection

GC tissues and paired adjacent normal tissues were obtained from 114 surgical patients between April 2014 and December 2017 at Ningbo First Hospital, China. The tissue samples were soaked immediately in RNA‐fixer Reagent (Bioteke, Beijing, China) after surgical removal from patients’ bodies and were stored at −80°C until analysis. Paired adjacent normal tissues were taken 5 cm from the cancer edge of GC and contained no obvious tumor cells, as assessed by two professional pathologists. No radiotherapy or chemotherapy was given to these patients before surgery. A total of 48 patients’ plasma samples were collected from peripheral veins before any treatment was applied. Based on age‐ and gender‐matching standards, 48 healthy people at Ningbo First Hospital, China, also had fresh plasma samples collected. All blood samples were treated with ethylenediaminetetraacetic acid (EDTA) as the anticoagulant and stored at −80°C until analysis as previously described.

Tumors were staged according to the Tumor‐Node‐Metastasis (TNM) staging system of the International Union Against Cancer. Histological grade was assessed according to the National Comprehensive Cancer Network (NCCN) Clinical Practice Guideline of Oncology (V.1.2011).

The Human Research Ethics Committee from Ningbo First Hospital approved all aspects of this study. All participants voluntarily joined this research with written informed consent before clinical trial in the study.

### 2.2 Cell culture

Normal human gastric mucosa epithelial cell lines (GES‐1) and human GC cell line AGS were purchased from the Shanghai Institute of Biochemistry and Cell Biology, Chinese Academy of Science (Shanghai, China). Another GC line MKN‐45 was purchased from the National Collection of Authenticated Cell Cultures. All cells were cultured in RPMI‐1640 medium (Life Technologies, Grand Island, USA) supplemented with 10% fetal bovine serum (Biochannel, Hangzhou, China) and cultured at 37°C in a humidified atmosphere with 5% CO_2_.

### 2.3 Total RNA extraction

The total RNA of the tissue samples and the cell samples was extracted by TRIzol reagent (Promega, USA) according to the manufacturer's protocol, whereas total RNA in plasma was extracted by TRIzol LS reagent (Promega). Then, the concentration and purity of total RNA were detected by a NanoDrop ND‐2000 spectrophotometer (Thermo Fisher Scientific, Wilmington, DE). Finally, RNA was stored at −80°C until analysis.

### 2.4 Reverse transcription

Total RNA samples were reverse‐transcribed into cDNA with random primers using the GoScript Reverse transcription system (Promega, Madison, USA) according to manufacturer‐provided protocols. The conditions of RNA reverse transcription were as follows: incubation at 25°C for 5 min, 42°C for 1 h, 70°C for 15 min and at 4°C for forever.

### 2.5 Polymerase chain reaction

Real‐time quantitative reverse transcription‐polymerase chain reaction (qRT‐PCR) was performed using GoTaq^TM^ 2‐step RT‐qPCR System (Promega) on the StepOnePlus QPCR Detection System (Life Technologies) following the manufacturer's instructions. Glyceraldehyde‐3‐phosphate dehydrogenase (GADPH) mRNA was used to normalize the levels of the circRNA. The sequences of hsa_circ_001888 divergent primers were as follows: Forward: 5′‐ TCCAGTGGCTCCCGTTTC‐3′ and Reverse: 5′‐ CGCCCAGCTTGCACTCA‐3′. The primers were synthesized by BGI Genomics (Guangdong, China). The reaction conditions were as follows: 95°C for 5 min for a not start, followed by 40 cycles at 95°C for 15 s, 60°C for 30 s and 72°C for 30 s. The C_q_ values were recorded for both hsa_circ_001888 and GADPH.

All reactions were performed in triplicate. The appearance of a single‐peak in the melt‐curve suggested the specificity of the PCR products. Relative quantification of gene expression was analyzed using ΔC_q_ method. All results are expressed as the mean ± SD.

### 2.6 Statistical analysis

Statistical analysis was performed with the Statistical Product and Service Solutions software 21.0 (SPSS, Chicago, IL, USA) and GraphPad Prism (GraphPad Software, La Jolla, CA, USA). The student t test and one‐way analysis of variance (ANOVA) were used to evaluate the differences between groups as appropriate. A receiver operating characteristic (ROC) curve was constructed to evaluate the diagnostic value of hsa_circ_001888. *p*‐values <0.05 were considered statistically significant.

## 3 RESULTS

### 3.1 Expression of hsa_circ_001888 in GC cell lines and tissues

**FIGURE 2:**
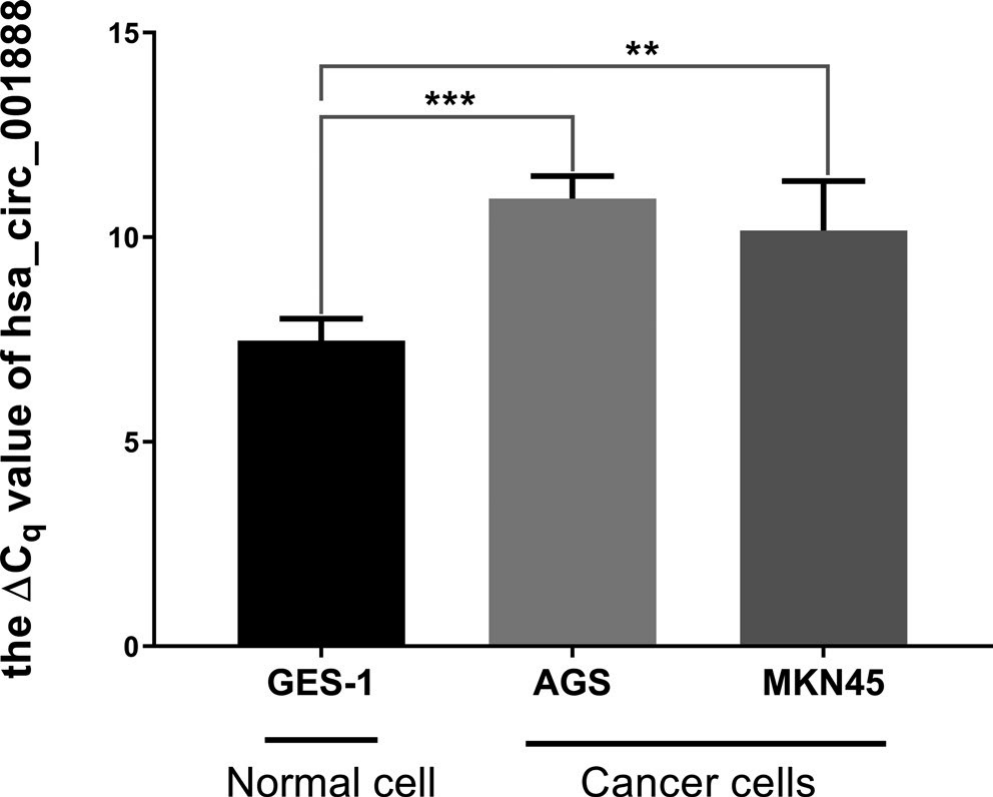
The relative expression of hsa_circ_001888 in gastric cancer cell lines. Hsa_circ_001888 expression levels in two human gastric cancer lines (AGS and MKN‐45) and normal gastric mucosa epithelial cell line (GES‐1) were determined by qRT‐PCR. Data are expressed as mean ± SD of three independent experiments. ** means *p* < 0.01, *** means *p* < 0.001

In this study, we first concluded that the sequence was completely consistent with that in CircBase. Then, we found that the expression levels of hsa_circ_001888 in human GC lines, AGS and MKN‐45, were reduced than the levels in the GES‐1 (Figure 2).

**FIGURE 3:**
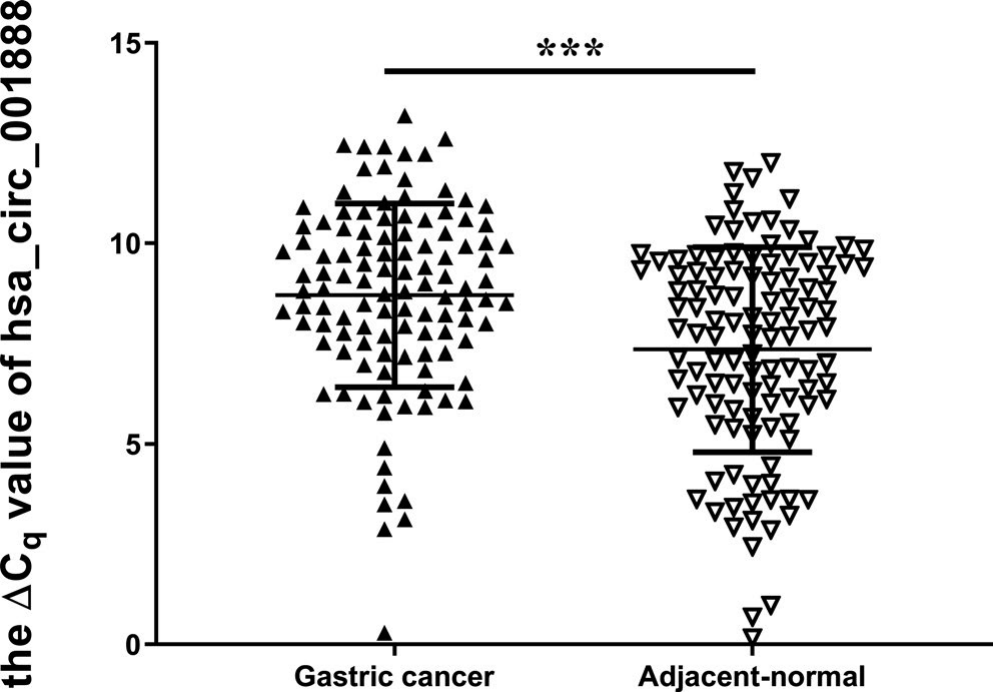
The expression levels of hsa_circ_001888 in gastric cancer tissues and adjacent normal tissues. Hsa_circ_001888 and GAPDH expression levels were detected by qRT‐PCR. The ΔCq method was used to analyze the relative expression of hsa_circ_001888. Data are expressed as mean ± SD of three independent experiments. *n* = 114, *** means *p* < 0.001

**FIGURE 4:**
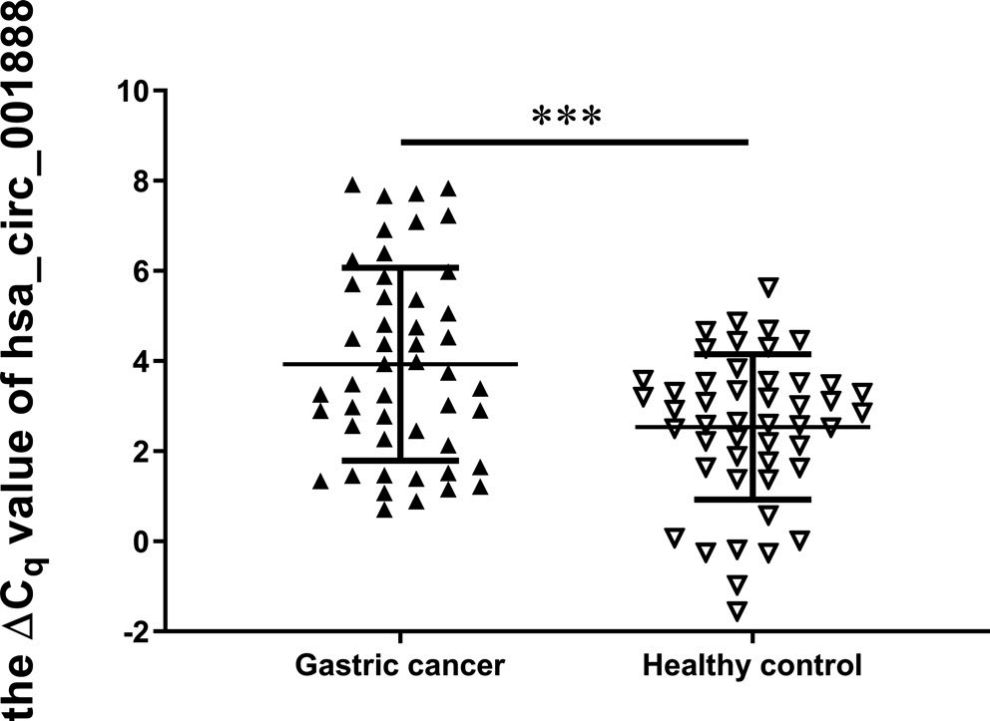
The expression levels of hsa_circ_001888 in gastric cancer plasma samples and healthy controls. Hsa_circ_001888 and GAPDH expression levels were detected by qRT‐PCR. Data are expressed as mean ± SD of three independent experiments. *n* = 48, *** means *p* < 0.001)

Furthermore, we explored hsa_circ_001888 expression levels in GC tissues and plasma samples of patients with GC. Similar to the results in the cell lines, our data suggested that hsa_circ_001888 was significantly decreased in GC tissues (*p* < 0.001, Figure 3). Its expression levels were significantly downregulated in 70.18% (80/114) GC tissues compared with the adjacent normal tissues. Moreover, hsa_circ_001888 levels in plasma samples of patients with GC were lower than those in healthy controls (*p* < 0.001, Figure 4). Its expression levels were significantly downregulated in 68.75% (33/48) GC plasma samples compared with healthy controls. The evidence suggested that hsa_circ_001888 was downregulated in either GC tissues or GC plasma samples compared to the control groups and may have the potential to be a biomarker for GC.

### 3.2 Relationships between hsa_circ_001888 expression levels and clinicopathological factors

Based on the above findings, we next analyzed the correlation between hsa_circ_001888 expression levels and the clinicopathological factors in patients with GC.

As shown in Table 1, the hsa_circ_001888 level in GC tissues was correlated with differentiation (*p* = 0.027). However, no association was found between its levels with other clinicopathological features, such as age, gender, invasion, carcinoembryonic antigen (CEA), carbohydrate antigen 19‐9 (CA19‐9) and lymphatic metastasis.

**TABLE 1:** The relationship of hsa_circ_001888 expression levels (ΔC_q_) in gastric cancer tissues with clinicopathological factors of patients with gastric cancer

Factors	No. of patients (%)	Mean ± SD	*p* value
Age (y)			
<60	27 (23.68)	7.86 ± 2.89	0.072
≥60	87 (76.32)	8.97 ± 2.01	
Gender			
Male	82 (71.93)	8.76 ± 2.43	0.687
Female	32 (28.07)	8.57 ± 1.91	
Diameter (cm)			
≥5	47 (41.23)	8.41 ± 2.18	0.250
<5	67 (58.77)	8.91 ± 2.35	
Differentiation			
Well + moderate	20 (0.18)	9.72 ± 1.59	**0.027** ^*^
Poor + undifferentiation	94 (0.82)	8.49 ± 2.36	
TNM Stage			
0&Ⅰ&Ⅱ	21 (0.18)	9.04 ± 2.46	0.463
Ⅲ&Ⅳ	93 (0.82)	8.63 ± 2.25	
Invasion			
Tis&T1	6 (0.06)	8.03 ± 2.11	0.458
T2&T3&T4	108 (0.94)	8.75 ± 2.30	
Lymphatic metastasis			
N0	19 (0.17)	9.08 ± 2.46	0.434
N1&N2&N3	95 (0.83)	8.63 ± 2.26	
Distal metastasis			
M0	105 (0.92)	8.68 ± 2.28	0.721
M1	9 (0.08)	8.97 ± 2.47	
Venous invasion			
Absent	38 (0.33)	8.91 ± 2.15	0.512
Present	76 (0.67)	8.61 ± 2.36	
Perineural invasion			
Absent	49 (0.43)	8.92 ± 2.36	0.394
Present	65 (0.57)	8.55 ± 2.23	
CEA			
Positive	31 (0.27)	8.52 ± 2.08	0.600
Negative	83 (0.73)	8.78 ± 2.37	
CA19‐9			
Positive	24 (0.21)	8.79 ± 1.39	0.797
Negative	90 (0.79)	8.69 ± 2.48	

Note

^*^ Bold indicates statistical significant value (*p* < 0.05).

As shown in Table 2, its expression level in GC plasma was linked with CEA (*p* < 0.001) and CA19‐9 (*p* = 0.036) expression. These results indicated that GC patients with positive CEA and CA19‐9 expression in plasma may have higher hsa_circ_001888 levels.

**TABLE 2:** The relationship of hsa_circ_001888 expression levels (ΔC_q_) in plasma from patients with gastric cancer and clinicopathological factors of patients with gastric cancer

Factors	No. of patients (%)	Mean ± SD	*p* value
Age (y)			
<60	14 (0.29)	4.32 ± 2.26	0.424
≥60	34 (0.71)	3.77 ± 2.11	
Gender			
Male	26 (0.54)	3.91 ± 2.49	0.952
Female	22 (0.46)	3.95 ± 1.71	
Diameter (cm)			
≥5	21 (0.43)	4.33 ± 2.06	0.259
<5	27 (0.56)	3.62 ± 2.19	
Differentiation			
Well + moderate	15 (0.31)	4.47 ± 2.02	0.241
Poor + undifferentiation	33 (0.69)	3.68 ± 2.18	
TNM Stage			
0&Ⅰ&Ⅱ	23 (0.48)	3.53 ± 2.10	0.213
Ⅲ&Ⅳ	25 (0.52)	4.30 ± 2.16	
Invasion			
Tis&T1	10 (0.21)	3.35 ± 2.26	0.339
T2&T3&T4	38 (0.79)	4.0 ± 2.12	
Lymphatic metastasis			
N0	19 (0.40)	3.68 ± 2.17	0.514
N1&N2&N3	29 (0.60)	4.10 ± 2.15	
Distal metastasis			
M0	47 (0.98)	3.85 ± 2.09	0.065
M1	1 (0.02)	7.83	
Venous invasion			
Absent	23 (0.48)	3.62 ± 1.96	0.346
Present	25 (0.52)	4.21 ± 2.30	
Perineural invasion			
Absent	29 (0.60)	3.75 ± 2.11	0.479
Present	19 (0.40)	4.20 ± 2.22	
CEA			
Positive	39 (0.81)	3.38 ± 1.89	**<0.001** ^*^
Negative	9 (0.19)	6.30 ± 1.50	
CA19‐9			
Positive	43 (0.90)	3.71 ± 2.08	**0.036** ^*^
Negative	5 (0.10)	5.82 ± 1.82	

Note

^*^ Bold indicates statistical significant value (*p* < 0.05).

### 3.3 ROC curve of hsa_circ_001888 level

**FIGURE 5:**
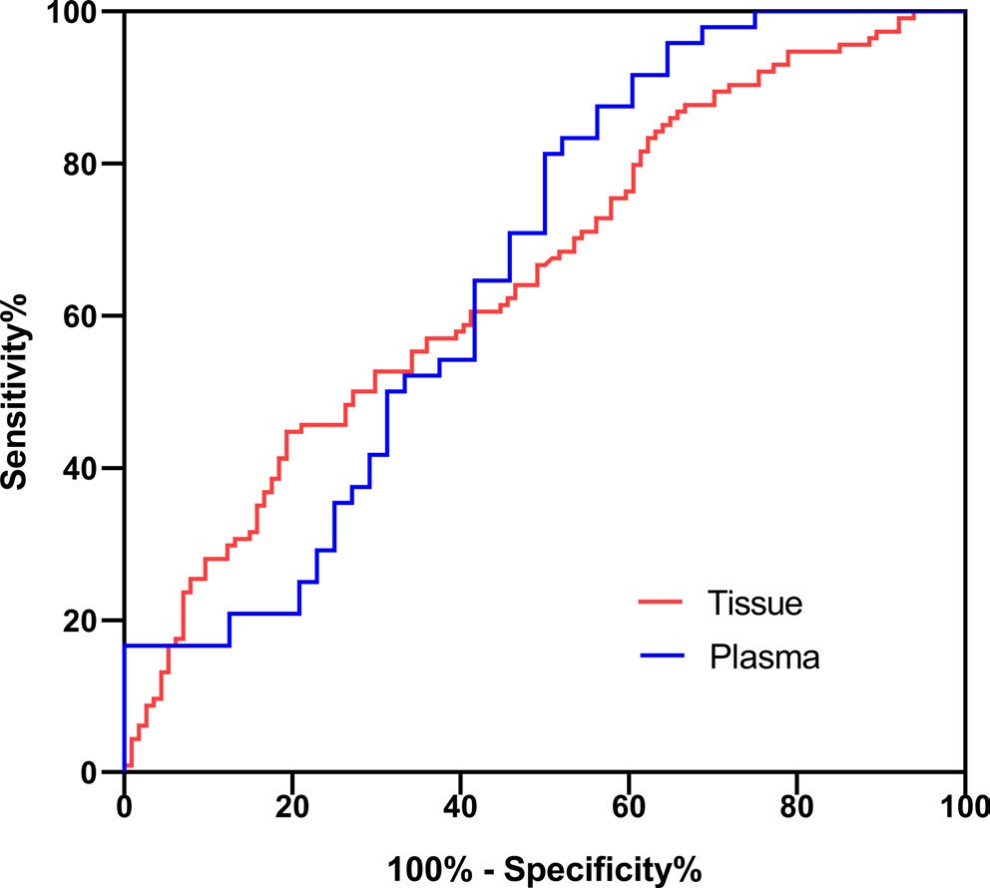
ROC curve of hsa_circ_001888

A ROC curve was established to explore the diagnostic values of hsa_circ_001888 (Figure 5). The area under the ROC curve (AUC) in GC tissues was 0.654. When the cut‐off value was 7.15, the specificity and sensitivity were 80.70% and 44.74%, respectively. The false‐negative rate and false‐positive rate were 55.26% and 19.30%, respectively.

The AUC of hsa_circ_001888 in plasma was up to 0.66. When the cut‐off value was 3.67, the specificity and sensitivity were 50.0% and 81.25%, respectively. The false‐negative rate and false‐positive rate were 18.75% and 50.0%, respectively.

## 4 DISCUSSION

CircRNAs are a novel family of endogenous RNAs characterized by a covalently closed‐loop structure. CircRNAs were first observed in RNA viruses as early as the 1970s. They were thought to be the by‐products of splicing errors at that time.^10^, ^11^ With the development of high‐throughput RNA sequencing technologies and bioinformatics methods, circRNAs were found to be extensively expressed in human cells and closely involved in the regulation of gene expression.^12^

Based on different originating mechanisms, circRNAs can be divided into three types: exonic circRNAs (ecircRNAs), circular intronic RNAs (ciRNAs) and extron‐intron circRNAs (eiciRNAs). Each type of circRNA can regulate gene expression. The formation of circRNAs involves the occurrence of direct back splicing or exon skipping.^4^, ^13^ CircRNAs have the significant properties that they are highly conserved and have a remarkably stable half‐life >48 h.^4^, ^14^ Therefore, circRNAs are regarded as useful biomarkers in diagnosing diseases.

Recent studies found that circRNAs containing miRNA response elements (MREs) can work as miRNA sponges.^14^, ^15^ For example, CiRS‐7 containing 74 miR‐7 binding sites can act as a miR‐7 sponge with high miRNA‐binding capacity and can participate in post‐transcriptional regulation.^5^ MiR‐7 plays a key role in Parkinson's disease, which indicates the potential role of CiRS‐7 in the progression of Parkinson's disease.^16^ As a super miRNA sponge, CiRS‐7 can act as an oncogene, promote tumor progression in various types of cancers, including GC.^17^, ^18^ Some circRNAs have the potential to be translated into proteins or peptides due to the presence of internal ribosomal entry sites (IRES) and open reading frames (ORFs).^19^ CircRNAs with protein translation functions may act as a novel type of protein‐coding RNA. Some circRNAs can interact with RNA‐binding proteins (RBPs) and regulated them during human epithelial‐mesenchymal transition (EMT).^20^ EMT is a critical cellular process in cancer metastasis and invasion.^21^

The function of circRNA in the diagnosis of GC also has been reported in recent years. Li et al.^22^ demonstrated the downregulation of hsa_circ_0001017 and hsa_circ_0061276 in GC tissues and plasma by RT‐droplet digital PCR (ddPCR). Zhang et al.^23^ reported that hsa_circ_0023642 was upregulated in GC and correlated with malignant progression of GC by acting as a miR‐223 sponge. Zhang et al.^24^ reported that circLARP4 can facilitate the expression of miR‐424‐5p target LATS1 and YAP by acting as a sponge of miR‐424‐5p. Another research displayed that hsa_circ_001649 restrained GC by sponging miR‐20a and then inactivated Wnt/β‐catenin and ERK pathways.^25^ CiRS‐7 was also reported to be significantly upregulated in GC tissues and linked to poor survival.^18^ CiRS‐7 can serve as a sponge of miR‐7, then decrease the expression of miR‐7‐mediated PTEN, increase the level of PI3K, AKT phosphorylation.^18^ Li et al.^26^ showed a downregulated circRNA in GC, Circ_104916, can inhibit cell proliferation, migration and invasion by decreasing the expression of Slug. The upregulation of E‐cadherin and downregulation of N‐cadherin, Slug, and Vimentin by western blot after Circ_104916 over‐expression, indicates the relationship of Circ_104916 in EMT.^26^

Although an increasing number of circRNAs have been identified and their potential functions have been researched, their diagnostic value remains largely unknown. In this study, hsa_circ_001888 was chosen as a targeted circRNA in GC because it is one of the circRNAs that may be associated with GC according to the bioinformatics analysis in the CircBase database. The results showed that hsa_circ_001888 were downregulated in two GC cell lines compared with GES‐1. These results are consistent with the bioinformatics analysis results. Then, we first explore hsa_circ_001888 expression levels in the tissues and plasma samples from various stages of gastric carcinogenesis. We explored hsa_circ_001888 was downregulated in GC tissues as well as in the plasma of patients with GC. Furthermore, we found the potential diagnostic value of hsa_circ_001888 in GC.

Differentiation is a crucial factor in the evaluation of the prognosis of GC.^27^ The results of the association between hsa_circ_001888 expression level and clinicopathological data showed that the expression of hsa_circ_001888 was more downregulated in poorly differentiated and undifferentiated tumors than in well‐differentiated ones (*p* = 0.011). The results indicated a close relationship between low expression levels of hsa_circ_001888 in GC and differentiation. However, further research will need to investigate the detailed molecular mechanisms of hsa_circ_001888 involved in GC progression. We next estimated the diagnostic value of hsa_circ_001888 in GC. By using ROC curve analysis, we found that in tissues, the AUC was 0.654; the specificity and sensitivity were 80.70% and 44.74%, respectively.

CEA and CA19‐9 are the most common and widely used plasma‐based biomarkers in the screening process for GC and other tumors in clinics. A study found that CEA and CA19‐9 positivity were significantly associated with depth of invasion, hepatic metastasis in GC.^28^ Tissue CEA expression is directly associated with long‐term prognosis.^29^ However, the positive rate of serum CEA and CA19‐9 in GC screening was low. Our research found that hsa_circ_001888 expression level was significantly associated with serum CEA (*p* < 0.001) and CA19‐9 expression (*p* = 0.036) levels. Ye et al.^30^ reported that hsa_circ_0001874 was downregulated in GC and correlated with the expression of CEA. Hsa_circ_0001895 was also related to CEA expression.^31^ The results indicate the possible interactive function of circRNA and CEA in GC diagnosing. By using ROC curve analysis, we found that in plasma, the AUC was 0.66; the specificity and sensitivity were 50.0% and 81.25%, respectively. Our data indicate that hsa_circ_001888 is a potential biomarker for GC with high degrees of accuracy.

The limitation of this study is that a relatively small sample size was used. More samples should be analyzed in future. The detailed molecular mechanisms of hsa_circ_001888 still need further research to explore.

In conclusion, our research implied that hsa_circ_001888 expression levels were significantly downregulated not only in GC tissues and cell lines but also in GC plasma samples. The expression of hsa_circ_001888 in GC tissues was associated with tumor cell differentiation. The decreased levels of hsa_circ_001888 in plasma of patients were significantly associated with serum CEA and CA19‐9 levels. Hsa_circ_001888 is a potential biomarker for the diagnosis of GC.

## CONFLICTS OF INTEREST

The authors declare that they are no conflicts of interest in this work.

## AUTHOR CONTRIBUTIONS

All authors equally contributed to this paper.

## Data Availability

The data used to support the findings of this study are available from the corresponding author upon request.
